# Protective Activity of Total Polyphenols from *Genista quadriflora* Munby and *Teucrium polium geyrii* Maire in Acetaminophen-Induced Hepatotoxicity in Rats

**DOI:** 10.3390/nu8040193

**Published:** 2016-04-01

**Authors:** Nacera Baali, Zahia Belloum, Samiya Baali, Beatrice Chabi, Laurence Pessemesse, Gilles Fouret, Souad Ameddah, Fadila Benayache, Samir Benayache, Christine Feillet-Coudray, Gérard Cabello, Chantal Wrutniak-Cabello

**Affiliations:** 1 UMR 866 Dynamique Musculaire et Métabolisme, Institut National de la Recherche Agronomique (INRA), Université de Montpellier, F-34060 Montpellier, France; nacirabek@hotmail.com (N.B.); chabi@supagro.inra.fr (B.C.); pessemes@supagro.inra.fr (L.P.); fouret@supagro.inra.fr (G.F.); cfeillet@supagro.inra.fr (C.F.-C.); gerard.cabello@orange.fr (G.C.); 2 Faculté des Sciences de la Nature et de la Vie, Département de Biologie Animale, Laboratoire de Biologie et Environnement, Université Constantine 1, 25000 Constantine, Algeria; baali-univc1@hotmail.com (S.B.); amedsouad@yahoo.fr (S.A.); 3 Unité de recherche (VARENBIOMOL), Département de Chimie, Faculté des Sciences Exactes, Université Constantine 1, 25000 Constantine, Algeria; mayanour2003@yahoo.fr (Z.B.); fbenayache@yahoo.fr (F.B.); sbenayache@yahoo.com (S.B.)

**Keywords:** acetaminophen, hepatotoxicity, *Genista quadriflora* Munby, *Teucrium polium geyrii* Maire, polyphenols, mitochondria, oxidative stress

## Abstract

Oxidative stress is a major cause of drug-induced hepatic diseases and several studies have demonstrated that diet supplementation with plants rich in antioxidant compounds provides a variety of health benefits in these circumstances. *Genista quadriflora* Munby (*Gq*) and *Teucrium polium geyrii* Maire (*Tp*) are known to possess antioxidant and numerous biological properties and these endemic plants are often used for dietary or medicinal applications. Herein, we evaluated the beneficial effect of rich-polyphenol fractions of *Gq* and *Tp* to prevent Acetaminophen-induced liver injury and investigated the mechanisms involved in this protective action. Rats were orally administered polyphenolic extracts from *Gq* or *Tp* (300 mg/kg) or *N*-acetylcysteine (NAC: 200 mg/kg) once daily for ten days prior to the single oral administration of Acetaminophen (APAP: 1 g/kg). The results show that preventive administration of polyphenolic extracts from *Gq* or *Tp* exerts a hepatoprotective influence during APAP treatment by improving transaminases leakage and liver histology and stimulating antioxidant defenses. Besides, suppression of liver CYP2E1, GSTpi and TNF-α mRNA levels, with enhancement of mitochondrial bioenergetics may contribute to the observed hepatoprotection induced by *Gq* and *Tp* extracts. The effect of *Tp* extract is significantly higher (1.5–2 fold) than that of *Gq* extract and NAC regarding the enhancement of mitochondrial functionality. Overall, this study brings the first evidence that pretreatment with these natural extracts display *in vivo* protective activity against APAP hepatotoxicity through improving mitochondrial bioenergetics, oxidant status, phase I and II enzymes expression and inflammatory processes probably by virtue of their high total polyphenols content.

## 1. Introduction

Human beings are exposed on a daily basis to toxic chemicals and pathogens, which cause serious health problems. Acetaminophen or paracetamol (*N*-acetyl-p-aminophenol; APAP) is one of the most extensively used analgesic and antipyretic agent worldwide. It is considered as a safe drug at normal therapeutic doses; however, its overdoses are known to produce acute hepatic necrosis, which may be fatal [1]. Development of liver injury by APAP is attributed to the formation of a reactive metabolic, *N*-acetyl-p-benzoquinoneimine (NAPQI), through oxidative metabolism mediated mainly by cytochrome P450 [2]. Generally, this product is detoxified through reaction with reduced glutathione (GSH); however, following a hepatotoxic dose of APAP, liver GSH levels are depleted and NAPQI covalently binds primarily to proteins cystein groups as 3-(cysteine-S-y) acetaminophen adducts [2]. Available data suggest that occurrence of mitochondrial dysfunctions may be an important mechanism involved in APAP-induced hepatotoxicity. Mitochondrial effects of APAP overdose have been recognized in rodents since the 1980s, when inhibition of mitochondrial respiration and depletion of ATP were first described [3,4]. More recent studies have shown the development of oxidative and nitrosative stress within mitochondria [5,6,7], and ultimately mitochondrial membrane depolarization and onset of the mitochondrial permeability transition [5,8,9].

Modulation of cellular thiols pool has been used as potential therapeutic strategies against APAP hepatotoxicity. When given with a loading dose of 140 mg/kg followed by 17 subsequent doses of 70 mg/kg every four hours, *N*-acetylcysteine (NAC) is regarded as a substance of choice for preventing alterations induced by APAP overdose in clinical practice [10]. As a precursor for hepatic GSH synthesis [1], it protects against cell injury by scavenging reactive oxygen and peroxinitrite inside mitochondria [7]. However, when used at higher doses, NAC can also produce adverse gastrointestinal events [11]. Therefore, NAC is safety under clinical treatment doses.

In the absence of reliable liver protective drugs in allopathic medical practices, searching for a novel and effective safe drug to prevent liver disorders remains an area of interest. Clinical research in this century has confirmed the efficiency of several plants in the treatment of liver diseases [12]. Natural products derived from plants such as polyphenols have received considerable attention in recent years due to diverse pharmacological properties, including antioxidant ability to counteract oxidative stress as observed in APAP-induced hepatic injury [13,14]. Antioxidants are considered as compounds acting by one or more of the following mechanisms: chemical reducing activity, free radical-scavenging, potential complexing of pro-oxidant metals and quenching of singlet oxygen [15]. Furthermore, they are able to increase endogenous antioxidant defenses and to modulate the cellular redox state [14,16].

The genus *Genista* L. (Fabaceae) occurs as 23 different species in Algerian flora, 11 of which are endemic. *Genista quadriflora* Munby (*Gq*) is distributed in Morocco (Rif and Middle Atlas) and North West of Algeria, in the Oran region [17,18]. Many of the plants belonging to the *Genista* genus are known to possess antioxidant and many other biological properties, ulceroprotective [19], anti-diabetic [20], estrogenic [21] and antiproliferative [22]. Phytochemical studies indicated that species of this plant contained various pharmaceutical active ingredients with antioxidant activity, among them flavonoids are predominant [19,20,22,23]. Quinolizidine alkaloids are also present in some species [24].

The genus *Teucrium* L. (Lamiaceae) displays an important natural diversity with more than 300 species mostly found in the Mediterranean region. In Algeria flora, this genus includes seven species and among them *Teucrium polium geyrii* Maire (*Tp*) named Takmazzut by the Touaregs [17,25]. Ethnobotanically, it is often used for preparation of tea and tonic and also as a spice plant [26,27,28]. In addition, it is traditionally considered as a nutriment for gastrointestinal function improvement in agreement with *in vitro* and *in vivo* demonstrated properties such as antispasmodic, antidiarrheal or ulcer protective activities [26,29,30,31]. Furthermore, numerous studies have proved antioxidant [32], anticancer [33] and hepatoprotective [34] activities of *Teucrium polium*. The benefit of *Teucrium* species is considered to be linked to the presence active compounds such as essential oils [35] and flavonoids [36] with biological and pharmacological activities.

Nevertheless, no reliable reports devoted to the knowledge of the mode of action of *Gq* and *Tp* against liver injury *in vivo* have been published, as far as we are aware. Keeping in mind plants literature and the possibility that free radicals and reactive oxygen species scavenging by natural antioxidants may protect tissues such as liver, the present study is planned to verify whether a pretreatment with polyphenolic extracts from *Gq* and *Tp* possesses protective effects against APAP-induced liver injury in rats and to explore action pathways involved in cytoprotection. The pretreatment procedure has been chosen from bibliographic data indicating that NAC pretreatment exerts a more efficient influence than NAC co or post treatments.

## 2. Materials and Methods

### 2.1. General Experimental Procedures

All reagents and chemicals applied in the study were of analytical grade.

### 2.2. Plant Material Collect and Extracts Preparation

*Genista quadriflora* Munby (*Gq*) and *Teucrium polium geyrii* Maire (*Tp*) were collected from their natural environnement and identified by Dr. Djamel Sarri (Département de la Biologie, Université de M’Sila, Algérie) and Dr. Ben Abdelhakim (Agence Nationale de Préservation de l’Environnement, Béchar, Algérie), respectively. Voucher specimens are stored at the Herbarium of the VARENBIOMOL research unit, University of Constantine 1.

*Genista quadriflora* Munby (*Gq*): The plant material is constituted of the aerial parts of *Gq*, collected from M’Sila in 2008. Aerial parts were dried (1130 g), and macerated with MeOH-H_2_O (80:20, v/v) for 24 h three times. The crude extract was concentrated at room temperature and diluted with 500 mL H_2_O. The remaining aqueous solution was extracted successively with petroleum ether, CHCl_3_, EtOAc and *n*-BuOH. The organic layers were dried with Na_2_SO_4_ giving after removal of solvents under reduced pressure, petroleum ether (0.3 g), CHCl_3_ (3 g), EtOAc (6 g) and *n*-BuOH (60 g) extracts, respectively.

*Teucrium polium geyrii* Maire (*Tp*): The plant material is constituted of the aerial parts of *Tp*, collected from the Mougheul region—Northeast of Bechar in 2007. The aerial parts were dried (494 g), and macerated with MeOH-H_2_O (70:30, v/v) for 24 h three times. The crude extract was concentrated at room temperature and diluted with 220 mL H_2_O. The remaining aqueous solution was extracted successively with petroleum ether, CHCl_3_, EtOAc and *n*-BuOH. The organic layers give after removal of solvents under reduced pressure, petroleum ether (0.3 g), CHCl_3_ (5.9 g), EtOAc (3 g) and *n*-BuOH (38.1 g) extracts, respectively.

Only the butanolic fractions of *Gq* and *Tp* were used to screen their biological effect in the present study. Indeed, n-Butanol allows a better extraction of polyphenols and concentrates polyphenolic compounds such as flavonoids, phenolic acids, tannins and anthocyanins [37].

### 2.3. Determination of Total Polyphenol Content

The total polyphenol content (TPC) of the *Gq* and *Tp* extracts was determined by spectrophotometry, using Folin-Ciocalteu reagent and gallic acid as standard (Sigma-Aldrich, St louis, MO, USA) as previously described [38]. Briefly, an aliquot of 100 µL of test extract was mixed with 250 µL of 1N Folin-Ciocalteau reagent for 2 min and 1250 µL of 20% Na_2_CO_3_ were then added. After 2 h of incubation at room temperature, the absorbance of reaction was measured at 760 nm using a spectrophotometer UV-120-02 (Shimadzu, Kyoto, Japan). The TPC of *Gq* and *Tp* extracts was expressed as Gallic acid equivalent concentration (mg GAE/g extract).

### 2.4. TLC-Fingerprint Analysis

The method was adapted from Sarr *et al.* [37]. *Gq* and *Tp* extracts were dissolved in the migration solvent of ethyl acetate/ethanol/formic acid/water (100:11:11:26). Twenty microliters of samples (0.35 mg/mL) were applied to the thin-layer chromatography (TLC) plate silica gel 60 F254 (Merck, Darmstadt, Germany). At the end of the migration, TLC plates were dried and phytochemical compounds were observed under natural light or under UV light in 366 nm after revelation by the Neu-reagent ((1% of diphenylboryloxyethylamine in methanol (from Sigma Chemicals Co., St. Louis, MO, USA)). Interpretation of the various chromatograms was made on the basis of those presented in Plant Drug Analysis [39]. Fluorescence was interpreted in the following way: blue, Phenolic Acids; yellow-orange, Flavonols; yellow-green, Flavones. For every specific spot of color with R_f_, an assignment was made with a type of compound, using the method described by Markham [40].

### 2.5. Animals

Male Wistar rats (Charles River, L’Arbresle, France) weighing 142 ± 8 g were used in this study. Rats were allowed to acclimate for one-week prior use and were housed in a controlled-temperature room with a 12-h light-dark cycle with unlimited access to standard food and water. Animal experiment was performed according to European directives (86/609/CEE) and approved by the Ethical Committee of Region Languedoc Roussillon, France (Ethical approval no: CE-LR-11008). Rats were randomized into five groups of six animals. Control rats and APAP intoxicated rats were orally administered with 5 mL/kg of 0.9% NaCl solution daily for ten days. NAC+APAP rats were orally treated with 200 mg/kg of *N*-acetylcystein (Sigma Co. St Louis, MO, USA) daily for ten days, according to Naglaa *et al.* [41]. *Gq*+APAP rats and *Tp*+APAP rats were orally treated with 300 mg/kg daily of *Gq* and *Tp* extracts respectively prepared in milli Q distil water for ten days on the basis of preliminary experiments demonstrating the efficiency of these doses. Comparison between low (300 mg/kg), medium (750 mg/kg) and high (1500 mg/kg) dose of extracts to protect against APAP toxicity was made. The dose of 300 mg/kg was chosen as the lower dose of extracts giving the best protection against APAP liver toxicity in our preliminary tests.

On the 11th day, apart those included in the control group, all rats received a single oral dose of acetaminophen (APAP; 1 g/kg). APAP was prepared from Doliprane tablets containing 500 mg of paracetamol (Sanofi-Aventis, France) as described by Nithianantham *et al.* [42]. In agreement with El-Shenawya *et al*. [43], 1 g/kg bodyweight APAP was given to induce hepatotoxicity in rats. Animals were anesthetized with sodium pentobarbital (50 mg/kg, *ip*) 24 h after APAP exposure. Blood was collected from abdominal vein into heparinized tube and allowed to clot. Plasma was separated by centrifugation at 1000 g for 10 min at 4 °C and frozen until analysis. Livers were removed immediately, weighed and washed with a cold sucrose buffer (0.25 mM sucrose, 10 mM Tris, 5 mM EDTA, pH 7.5). Samples were used for histological study, for isolation of mitochondria or snap frozen in liquid nitrogen and stored at –80 °C for further analysis.

### 2.6. Biochemical Analysis

Plasma aspartate aminotransferase (AST) and alanine aminotransferase (ALT) were determined using biochemical micro-assays and COBA-MIRA^+^ automatic analyzer (Platform Anexplo/Genotoul, Toulouse, France).

### 2.7. Histological Analysis

Liver samples were fixed in 10% formalin for 24 h and embedded in paraffin. Microtome sections of 5 µm thickness were prepared and stained with haematoxylin-eosin prepared according to the standard procedure of RHEM (Réseau d’Histologie Expérimentale de Montpellier, France). Histopathological liver sections were observed using an Exacta+Optech microscope (GmbH, München, Germany) fitted with a digital camera (Canon DS126181, Tokyo, Japan).

### 2.8. Oxidative Stress Markers

Frozen liver tissues were homogenized in ice cold phosphate buffer 50 mM, pH 7.0 using Ultra Turax homogenizer and processed for the measurement of TBARs levels as an index of lipid peroxidation [44]. A part of the homogenate was treated with 10% metaphosphoric acid for the estimation of reduced glutathione using the method of Griffith (1980) [45]. The remaining homogenate was centrifuged at 3000 rpm for 10 min and the obtained supernatant subsequently used for superoxide dismutase (SOD), glutathione peroxidase (GPx), glutathione reductase (GRx) and glutathione transferase (GST) activity measurements. SOD, GPx, GRx and GST activities were measured according to Marklund and Marklund (1974), Flohé and Günzler (1984), Carlberg and Mannervik (1985) and Habig *et al.* (1974), respectively [46,47,48,49].

### 2.9. Mitochondria Isolation

Liver mitochondria were isolated as previously described by Frezza *et al*. [50]. Briefly, a sample of about 2 g of liver was homogenized on ice in a ratio 1 g wet tissue for 10 volumes of sucrose buffer (0.25 mM sucrose, 10 mM Tris Base, 5 mM EDTA, pH 7.5) using a motor-driven/Teflon Potter Elvehjem homogenizer. The homogenate was centrifuged at 900 g for 10 min at 4 °C. The resulting supernatant fraction was centrifuged at 10,000 g for 10 min at 4 °C. The pellet was suspended in sucrose buffer and centrifuged at 10,000 g for 10 min at 4 °C. The final mitochondrial pellet was suspended in a minute volume of respiratory medium (MIRO5 medium: 0.5 mM EGTA, 3 mM MgCl_2_, 60 mM K-Lactobianate,20 mM Taurine,10 mM HK_2_PO_4_, 20 mM HEPES, 110 mM sucrose and 1 g/L BSA, pH 7.4), and kept on ice until assayed. Unused mitochondria were frozen and stored at –80 °C until needed.

### 2.10. Mitochondrial Respiration

Mitochondrial oxygen consumption was measured using the high resolution Oxygraph-2K (OROBOROS instruments, Innsbruk, Australia). In two sealed thermostated chambers (37 °C) with continuous stirring at a constant temperature of 37 °C, freshly isolated mitochondria (200 µg protein) were incubated in 2 mL of the respiratory medium MIRO5 (0.5 mM EGTA, 3 mM MgCl_2_·6H_2_O, 65 mM KCl, 20 mM taurine, 10 mM KH_2_PO_4_, 20 mM HEPES, 110 mM sucrose, and 1 g/L BSA, pH 7.1). State 4 respiration (resting) was initiated by adding 5 mM glutamate and 2.5 mM malate. Subsequent addition of 0.5 mM ADP generated glutamate and malate supported state 3 (ADP stimulated respiration). Data acquisition and analysis was performed using Oxygraph-2K-DataLab software version 4.3.2.7 (OROBOROS instruments, Innsbruk, Austria). Respiratory control ratio (RCR) was determined as the ratio between oxygen consumption in state 3 and state 4 [51].

### 2.11. Mitochondrial Respiratory Complexes and Citrate Synthase Activities

The maximal enzymatic activity of mitochondrial respiratory chain complexes (CI, CII, CII+III, CIV) and citrate synthase (CS) were measured in isolated liver mitochondria. CI (NADH-ubiquinone oxidoreductase) activity was measured spectrophotometrically by following 2, 6-dichloroindophenol (DCIP) reduction by NADH at 600 nm according to Janssen *et al.* [52]. C II (succinate ubiquinone oxidoreductase) activity was determined spectrophotometrically by following the reduction of DCIP by succinate at 600 nm [53]. CII+III (succinate cytochrome C reductase) activity was measured spectrophotometrically by following the rate of reduction of cytochrome C at 550 nm as described by Rustin *et al.* [53]. CIV (cytochrome c oxidase) activity was measured spectrophotometrically by following the oxidation of reduced cytochrome c at 550 nm as described by Wharton and Tzagoloff [54]. CS activity was determined as the rate of color change of 5, 5-dithiobis-2-nitrobenzoic acid (DNB) at 450 nm according to Srere [55].

### 2.12. Western Blot Analysis

Livers were homogenized using a Polytron homogenizer in a Tris-NP40 buffer (50 mM Tris, pH 8.0, 150 mM NaCl, 1% Nonidet P-40) supplemented with a protease inhibitor cocktail (Roche Diagnostics). The homogenates were incubated on ice for 10 min and centrifuged at 10000 g for 10 min to remove tissue debris. Fifty µg of proteins were run on SDS-PAGE mini-gels at the appropriate concentration of acrylamide and transferred onto a polyvinylidene difluoride membrane. Membranes were blocked (1 h at room temperature) with a 5% skim milk in 1×TBST (Tris-buffered saline Tween-20: 20 mM Tris-HCl, pH 7.6, 137 mM NaCl, and 0.2% Tween-20) solution and probed with an antibody raised against CYP2E1 (1:1000 dilution, rabbit polyclonal ab84598, (R, H), Abcam, Cambridge, UK) or ß-actin (1:200 dilution, rabbit polyclonal sc-81178 (H, M, R, Hm) Santa Cruz Biotechnology INC, Dallas, TX, USA) overnight at 4 °C. After washing with TBST, blots were incubated at room temperature (1 h) with the appropriate secondary antibody coupled to horseradish peroxidase and washed again. Antibody-bound protein was revealed using the ECL reagent (Thermo Scientific). Films were scanned and analyzed using Image J software. All blots were corrected for loading using ß- actin expression.

### 2.13. Analysis of mRNA Levels by Real-Time PCR

Total RNA was isolated from liver tissue using the Trizol reagent (Invitrogen Life Technologies) as recommended by the manufacturer. mRNA gene expression was determined by Real-time Quantitative Polymerase Chain Reaction (qPCR). One microgram total RNA was reverse-transcribed using SuperScript™ First-strand synthesis system, with 50 units of Superscript™ II reverse transcriptase, random hexamers and Oligo (dT) primers (Invitrogen Life Technologies) according to the manufacturer’s instructions. Reverse transcription was performed simultaneously for all samples. Real-time PCR analyses were performed in a Mini Opticon detection system (BioRad, Hercules, CA, USA) with 8 µL of IQ^TM^ SYBR Green Supermix (Biorad, Hercules, CA, USA), 200 nM of both Forward and Reverse primers of target genes (CYP2E1, GSTpi and TNF-α), 2 µL of cDNA template and water to a final volume of 16 µL. Gene specific primers for target genes were designed using Primer Express Software (CYP2E1 forward: 5′-TTCCAACCTACCCCATGAAG-3′; reverse: 5′-GAGGGAGTCCAGAGTTGGAA-3′), (GSTpi forward: 5′-GCCATCTTGAGGCACCTG-3′; reverse: 5′-CACCCCATCATTCACCATATC-3′) and (TNF-α forward: 5′-TGAACTTCGGGGTGATCG-3′; reverse: 5′-GGGCTTGTCACTCGAGTTTT-3′). Normalization was performed from simultaneous amplification of a ß-actin gene fragment (forward: 5′-AATCCTGTGGCATCCATGAAAC-3′; reverse: 5′-CGCAGCTCAGTAACAGTCCG-3′). The real time PCR conditions were as follow: after an initial denaturation step for 3 min at 95 °C, 40 cycles of 95 °C for 10 s and 60 °C for 30 s. Melting point dissociation curves were performed between 65 °C and 95 °C (temperature transition of 0.5 °C) to confirm that only a single product was amplified. To ensure quality of the measurements, each PCR experiment for each gene included a negative control (sample replaced by RNase free water). Results were expressed using the comparative cycle threshold (Ct) method (CFX Manager, Biorad). The ΔCt values were calculated in every sample for each gene of interest as followed: Ct of gene of interest minus Ct of reporter gene with ß-actin as the reporter gene.

### 2.14. Protein Levels

All protein concentrations were determined using a Bradford assay (Bio-Rad, Marnes-la-Coquette, France).

### 2.15. Statistical Analysis

Experimental results are presented as means ±SD. A Student’s *t* test was used to compare the total polyphenol content between the n-BuOH extracts from Gq and Tp. For the other experiments, statistical analyses were performed using a one way analysis of variance (ANOVA) followed by a Fisher’s test using the statistical package GraphPad Prism. For all tests, the statistical significance was set at *p* < 0.05.

## 3. Results

### 3.1. Total Polyphenols Content (TPC) and TLC -Fingerprint of Gq and Tp Extracts

The Folin–Ciocalteu assay is one of the oldest methods developed to determine the content of total phenols [38]. The total polyphenol content (TPC) found in n-BuOH extracts was significantly lower for *Gq* (228 ± 5 mg GAE/g of extract) than for *Tp* (251 ± 4 mg GAE/g of extract, Student’s *t*: *p* < 0.01).

TLC is one of the numerous methods used to provide a chromatographic plant extract fingerprint. The reagent of Neu has been used to detect flavonoids. This reagent, indeed, reveals them as colorful stains in blue, orange, green, red and yellow fluorescence [37]. Twelve spots lights were detected in *Gq* extract (Table 1) corresponding to phenolic acids (band N°5, 10, 11 and 12), flavonoids (band N°1, 2, 3, 4, 6, 7 and 8) and not identified compounds (band N°9). Nine spots lights were detected in *Tp* extract (Table 1) corresponding to phenolic acids (band N°1′, 3′ and 9′), flavonoids (band N°3′, 4′, 5′ and 6′) and not identified compounds (band N°2′, 7′ and 8′). These chromatograms indicated that *Gq* and *Tp* extracts contain phenolic acids and flavonoids of interest. Among the flavonoid subclasses, flavonols, flavones, isoflavones, flavonones, flavonol glycosides are the most widespread in both extracts. However, flavonoid aglycones and methylated flavones were detected in Gq extract. Phenolic acids are detected in both extracts. Overall, flavonoids and phenolic acids are predominant polyphenolic compounds in *Gq* and *Tp* extracts, respectively.

### 3.2. Influence of Gq and Tp Extracts on Blood Transaminases Levels

**Figure 1:**
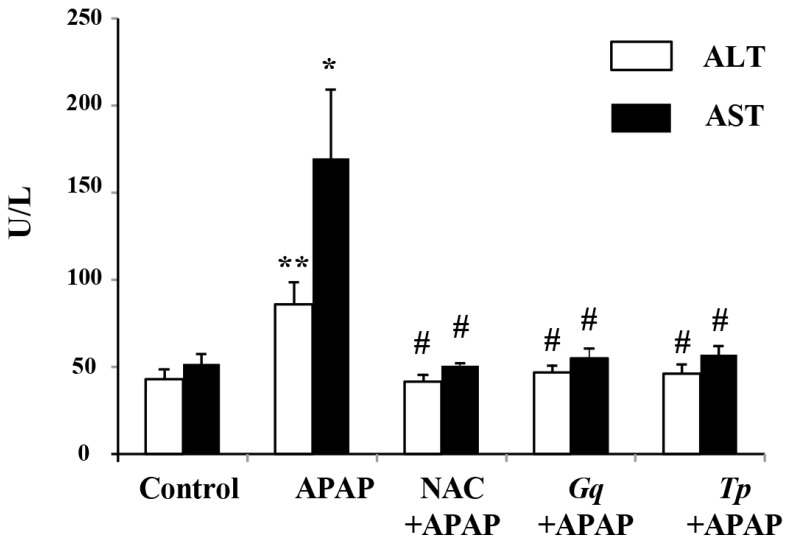
Effect of *Gq* or *Tp* extract on plasma alanine aminotransferase (ALT) and aspartate aminotransferase (AST) levels following APAP toxicity. Results are means ± SD (*n* = 6). One-way ANOVA followed by Fisher’s test: * *p* < 0.05 and ** *p* < 0.01 *vs.* control group; # *p* < 0.05 *vs.* APAP group.

The serum levels of hepatic enzymes AST and ALT, used as biochemical markers for evaluation of early hepatic injury, were significantly higher (+100%; *p* < 0.01 and +227%; *p* < 0.05 respectively) in APAP-treated animals than untreated animals (Figure 1). The 300-mg/kg daily pretreatment with *Gq* or *Tp* extract significantly prevented the elevation of these marker enzymes (*p* < 0.05; Figure 1), observed in APAP-treated group. NAC, a reference drug to prevent hepatic injury, exerted a quite similar influence.

### 3.3. Influence of Gq and Tp Extracts on Liver Histology

**Figure 2:**
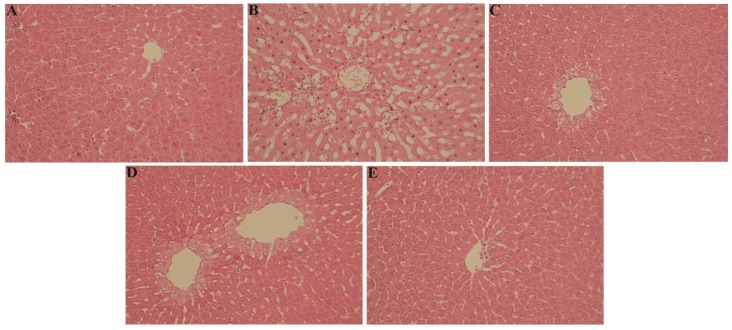
Effects of *Gq* or *Tp* extract on liver histological changes following APAP toxicity in rats: (**A**) Control group; (**B**) APAP treated group; (**C**) NAC pretreated group; (**D**) *Gq* pretreated group; and (**E**) *Tp* pretreated groups (hematoxylin–eosin, 100X).

When compared to liver histological microphotographs from control rats (Figure 2A), liver sections of APAP-treated group exhibited an obvious disarrangement of hepatic cells with intense centrilobular necrosis, sinusoid dilatation and inflammatory cells infiltration (Figure 2B). Liver sections of APAP-treated animals receiving either *Gq* extract or NAC showed reduced disarrangement of hepatic cells with hepatocytes degeneration only restricted to cells surrounding the centrilobular vein; many lobules were not affected, indicating a marked hepatotoxicity prevention of *Gq* extract or NAC pretreatment (Figure 2C,D). Interestingly, pretreatment with *Tp* extract fully abrogated the histopathological abnormalities associated to APAP overdose (Figure 2E).

### 3.4. Effect of Gq and Tp Extracts on Liver Oxidative Stress Markers

The oxidative stress, considered as a major mediator of APAP-induced liver damage, was assessed by measuring the activity of hepatic antioxidant defense enzymes (SOD, GPx, and GR), GSH and the level of lipid peroxidation products (TBARs). APAP administration markedly increased TBARs levels (+64%), reduced GSH levels (−56%) and antioxidant enzymes activities (SOD: −21%, GPx: −41%, GR: −26%; *p* < 0.001 for all) when compared with the control group (Table 2). Pretreatment with either *Gq* or *Tp* extract induced a significant decrease in TBARs levels (−36% and −33%, respectively) and enhanced functional antioxidant markers (SOD, GPx, GR and GSH) after APAP treatment, relatively to animals without pretreatment (all at *p* < 0.001; Table 2). The improvement exhibited by *Gq* and *Tp* extracts was similar to that induced by NAC. However, *Gq* and *Tp* extracts pretreatments also significantly increased GR activity by 21% and 14%, respectively, relatively to APAP untreated control animals (respectively, *p* < 0.001 and *p* < 0.05).

### 3.5. Effect of Gq and Tp Extracts on Liver Mitochondrial Activity

Several studies have reported that mitochondrial dysfunctions are observed in APAP-induced liver injury. In this work, we observed that APAP inhibited mitochondrial respiration rate (state 4: −59%; *p* < 0.001 and state 3: −80%; *p* < 0.01; RCR: −51%; *p* < 0.05; Table 3) with concomitant decreases in Complex I (CI: −27%; *p* < 0.01), Complex II (CII: −26%; *p* < 0.05) and citrate synthase (CS: −19%; *p* < 0.01) maximal activities (Table 3). Pretreatment with NAC strongly increased state 4 (+84%; *p* < 0.01) and state 3 (+184%; *p* < 0.05) mitochondrial respiratory rate measured after APAP administration in as well as CS activity (+16%; *p* < 0.05, Table 3). However, NAC pretreatment did not significantly affect mitochondrial complexes activities in APAP treated animals (Table 3). Interestingly, in these rats, *Tp* extract exhibited a pronounced effect on mitochondrial respiratory rate (state 4: +96% *p* < 0.01; state 3: +324% *p* < 0.001; RCR: +116% *p* < 0.05; Table 3) and prevented the decrease of all measured mitochondrial respiratory complexes and CS maximal activities occurring after APAP treatment (relatively to not pretreated animals: CI: +79% *p* < 0.001; CII: +88% *p* < 0.001; CIV: +36% *p* < 0.01; CS: +48% *p* < 0.01; Table 3). In contrast, pretreatment with *Gq* extract exerted only a significant improvement of mitochondrial respiratory complexes and CS maximal activities (CI: +63% *p* < 0.001; CII: +105% *p* < 0.001; CIV: +46% *p* < 0.01; CS: +42% *p* < 0.01; Table 3) without any influence on respiratory activity. Even more interesting is the observation that, in *Tp* extract pretreated rats, the maximal activities of CI, CII and CIV were significantly higher (+31%, +39% and +33%, respectively; *p* < 0.01) than in APAP untreated control animals. The same influence of *Gq* extract pretreatment on CII (+52%) and CIV (+42%) activities both at *p* < 0.01 was observed.

### 3.6. Effect of Gq and Tp Extracts on Liver CYP2E1 Protein and mRNA Levels

**Figure 3:**
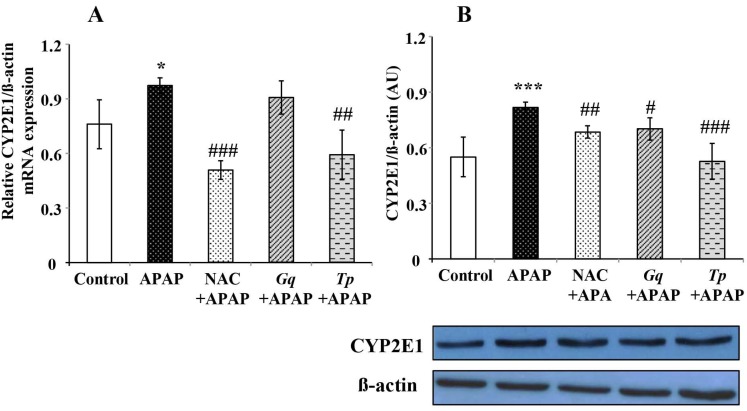
Effect of *Gq* or *Tp* extract on liver phase I enzyme following APAP toxicity in rats: (**A**) CYP2E1 mRNA expression levels; and (**B**) CYP2E1 protein levels. AU = arbitrary unit. Results are means ± SD (*n* = 6). One-way ANOVA followed by Fisher’s test: * *p* < 0.05 and *** *p* < 0.001 *vs.* control group; ^#^ *p* < 0.05, ^##^ *p* < 0.01 and ^###^ *p* < 0.001 *vs.* APAP group.

To determine whether pretreatment with *Gq* or *Tp* extract affected APAP metabolism, we check CYP2E1 levels, as this phase I enzyme catalyzes APAP conversion to hepatotoxic NAPQI. APAP treatment significantly increased CYP2E1 mRNA (+28%, *p* < 0.001; Figure 3B) and protein levels (+49%, *p* < 0.05; Figure 3A) relatively to untreated animals, confirming the induction of the CYP2E1 isoform by APAP. Interestingly, this increase in CYP2E1 protein and mRNA levels was markedly decreased by *Tp* extract (respectively −35%, *p* < 0.001 and −39%, *p* < 0.01) or NAC pretreatments (respectively −16%, *p* < 0.01 and −47%, *p* < 0.001) as shown in Figure 3. Pretreatment with *Gq* extract also reduced CYP2E1 protein levels increase after APAP (−15%, *p* < 0.05; Figure 3B), without influence on mRNA levels (Figure 3A).

### 3.7. Effect of Gq and Tp Extracts on Liver GST activity and GSTpi mRNA Levels

**Figure 4:**
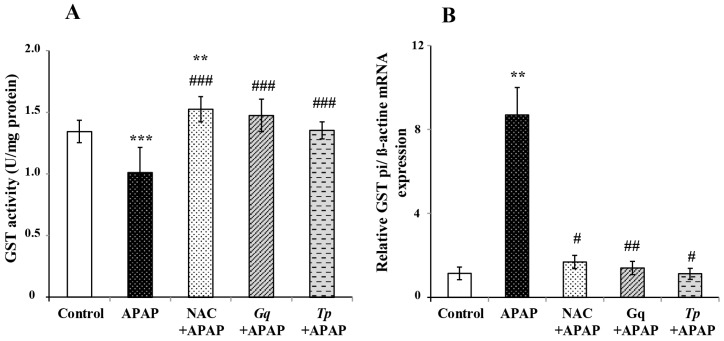
Effect of *Gq* or *Tp* extract on liver phase II enzyme following APAP toxicity in rats: (**A**) GST activity; and (**B**) GSTpi isoform mRNA expression levels. Results are means ± SD (*n* = 6). One-way ANOVA followed by Fisher’s test: ** *p* < 0.01 and *** *p* < 0.001 *vs.* control group; ^#^ *p* < 0.05, ^##^ *p* < 0.01 and ^###^ *p* < 0.001 *vs.* APAP group.

GST, a phase II enzyme of drug metabolism, catalyzes the conjugation of reactive metabolites with GSH. We found that in APAP treated rats, GST activity was reduced (−25% *p* < 0.01; Figure 4A) whereas GSTpi mRNA levels were found dramatically increased (+7.5 fold *p* < 0.01; Figure 4B). Pretreatment with NAC, *Gq* or *Tp* extract significantly enhanced GST activity (+50%, +45%, +34%, respectively, all at *p* < 0.001; Figure 4A) when compared to the APAP treated group. Interestingly, the three pretreatments markedly blunted the increase of GSTpi mRNA expression (*p* < 0.05, *p* < 0.01 and *p* < 0.05, respectively; Figure 4B) induced by APAP. In addition, only NAC increased GST activity (+13% *p* < 0.01; Figure 4A) when compared to control APAP untreated rats.

### 3.8. Effect of Gq and Tp Extracts on Liver TNF-α Expression Levels

**Figure 5:**
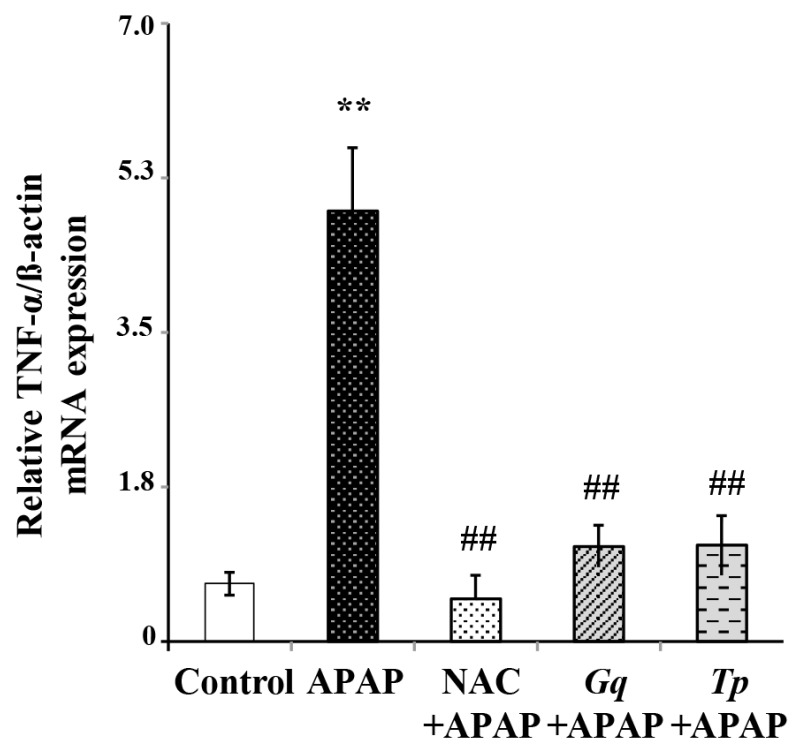
Effect of *Gq* or *Tp* extract on Liver TNF-α mRNA levels following APAP toxicity. Results are means ±SD (*n* = 6). One-way ANOVA followed by Fisher’s test: ** *p* < 0.01 *vs.* control group; ^##^ *p* < 0.01 *vs.* APAP group.

The increased susceptibility to APAP liver injury has been reported to correlate with an elevated expression of liver pro-inflammatory cytokines such as TNF-α. Therefore, we have observed the effects of *Gq* and *Tp* extracts on mRNA expression levels of this cytokine (Figure 5). APAP administration significantly up-regulated TNF-α mRNA expression (+7.4 fold; *p* < 0.01) relatively to control animals, suggesting induction of a severe inflammatory response, which may be influenced by the concomitant oxidative stress status previously found. Moreover, pretreatment with *Gq* extract, *Tp* extract or NAC markedly blunted the increase of TNF-α mRNA expression (for all, *p* < 0.01) during APAP treatment.

## 4. Discussion

Crude extracts of medicinal plants have received increasing interest for prevention of diet or drug induced pathologies, due to protection conferred by presence in significant amounts of components with antioxidant activity. It has been recognized that the polyphenol content of plant extracts is a main part of their antioxidant activities due to their redox properties, allowing them to act as reducing agents, hydrogen donors and singlet oxygen quenchers [15]. In this study, we have focused our attention on *Genista quadriflora* Munby (*Gq*) and *Teucrium polium geyrii* Maire (*Tp*). Our data indicate that both *Gq* and *Tp* extracts have higher amounts of polyphenols when compared to *Genista vuralii* (212 mg GAE/g extract) [23] or *Teucruim poluim L*.subsp.*polium* (158 mg GAE/g extract) [27], in agreement with the fact that polyphenols amounts and chemical composition are affected by different factors, such as genotype, environmental conditions and extraction procedures [27,56].

To better identify the polyphenolic compounds of *Gq* and *Tp* extracts (phenolic acids, flavonoids) whose antioxidant and hepatoprotective effects have already been the subject of numerous studies, we proceeded to a TLC-fingerprint analysis of the *Gq* and *Tp* extracts. Presence of flavonoids and phenolic acids as the predominant compounds in *Gq* and *Tp* extracts, respectively, may contribute to the high TPC of these species. These results have been confirmed by HPLC chromatogram analyses of *Gq* and *Tp* extracts (ZB, data not shown).

As the antioxidant potential of polyphenolic compounds in plant belonging to the genus *Genista* and *Teucrium* has been reported in a number of *in vitro* studies [22,23,32,36,57], these results prompted us to screen antioxidant activity and hepatoprotective effects of polyphenolic extracts from *Gq* or *Tp* in rats submitted to APAP overdose.

Several experiments have previously demonstrated that plant derived phenolic compounds exert potent antioxidative properties when given before APAP administration [58,59]. Moreover, comparison of kaempferol treatment, cotreatment or posttreatment indicated that the protective influence of this flavonol on doxorubicine induced cardiotoxicity occurred only when it was given before doxorubicine administration [60]. Consequently, in this first study, we have chosen to test the influence of *Gq* or *Tp* extract pretreatment on APAP hepatotoxicity. NAC was used as a positive control in the same experimental conditions. No consequences of *Gq* extract, *Tp* extract or NAC administration on rat bodyweight and no pathological signs were observed during the 10 days pretreatment period.

Serum enzyme levels such as aspartate transaminase (AST) and alanine transaminase (ALT) are commonly used as hepatic markers to assess APAP-induced liver damage [61]. As expected, in the present study, APAP treatment (1 g/kg) significantly increased the serum levels of these hepatic enzymes. These changes reflected the occurrence of APAP-induced hepatocellular damages appearing from histological data demonstrating disarrangement of hepatic cells with intense centrilobular necrosis, sinusoid dilatation and inflammatory cells infiltration in APAP-treated animals. Interestingly, a preventive treatment by *Gq* extract (300 mg/kg), *Tp* extract (300 mg/kg) or NAC (200 mg/kg) used as a positive antioxidant control, fully abrogated the APAP-induced increase in hepatic serum enzymes, suggesting a stabilization of hepatic membranes. These biochemical findings were in agreement with our histological data demonstrating a reduction (*Gq* or NAC) or a full abrogation (*Tp*) of structural liver abnormalities. Similarly, a beneficial effect of *Teucruim* spiece on AST and ALT release and liver histology, has been reported in rat liver cancer and APAP toxicity in mice [33,34]. In the same line, Yousef *et al.* [14] have reported that NAC, curcumin or quercetin normalizes transaminases levels and restores liver histology in APAP-intoxicated rats, thus suggesting that antioxidant compounds occurring in *Gq* or *Tp* extract are involved in the beneficial influence of the extracts observed in our study.

Oxidative stress is a major mechanism underlying the pathogenesis of APAP-induced liver damage [6,62]. An overdose of APAP saturates detoxification pathways, leading to hepatic GSH depletion and excessive production of NAPQI, which freely binds to cellular molecules [2]. Lipid peroxidation is an oxidative modification of unsaturated lipids and is involved in the destructive processes that affect liver in APAP overdose [63,64]. The current work substantiates that ROS generated by APAP administration may be responsible for the observed increased lipid peroxidation rate and altered antioxidant status. GSH plays an essential role in detoxification of NAPQI and prevention of APAP induced liver injury [8,62]. Because NAPQI is directly detoxified by GSH, the fall in hepatic GSH levels probably reflects its intensive use in this process. In addition, we observed that the GSH replenishing (GR) and GSH depleting (GPx, and GST) enzymes activities were inhibited in APAP treated rats, thus affecting the conversion of GSSH to GSH and the radical scavenging capacity of cells. Furthermore, the simultaneous decrease in SOD activity also significantly contributed to the reduction in antioxidative capacity inducing a stronger degradation of the cell status.

The antioxidant properties of *Gq* and *Tp* extracts pretreatments on APAP toxicity are clearly related to restoration of the GSH level and of antioxidant enzymes activity. Phytoconstituents derived from plants have gained much importance recently due to their diversified biological properties including antioxidant and hepatoprotective activity which could be provided by both pretreatment (prophylactic) and posttreatment (curative) [13,14,34]. It has been reported that a number of antioxidant plants have shown to be promising in protecting against APAP-induced liver injury [13,14]. The cytoprotection provided by pretreatment with our plant extracts may be due to (1) their interference with the metabolic activation of APAP by CYP2E1 enzymes, (2) their interference with the binding of NAPQI to cellular proteins at the initial steps of APAP toxicity, or (3) their antioxidant properties by directly scavenging intracellular ROS [14,15]. This influence was at least as efficient than that of the standard NAC antioxidant, classically used as a known APAP antidote acting by promoting liver GSH synthesis [1]. Previous studies demonstrated that flavonoids could stimulate the the activities of liver antioxidant enzymes that are responsible for intracellular GSH synthesis [14,65,66]. Thus, the mechanism of the hepatoprotective action of rich-polyphenol fraction from both *Tp* and *Gq* may be, in part, due to enhancement of intracellular glutathione levels. In this regard, improvement of GSH pathways is an important part of the *Teucruim* species polyphenolic antioxidative influence [16,57].

It is widely accepted that mitochondrial dysfunction is associated to APAP induced liver injury. In this work, we observed that APAP inhibited mitochondrial respiration with concomitant decreases in respiratory complex I and II and citrate synthase specific activities. As citrate synthase activity is also a marker of mitochondrial mass, our results established that mitochondrial mass and biochemical activity are significantly altered by APAP. These abnormalities could be explained on the basis of previous findings indicating sensitivity of protein sulfhydryls in mitochondrial respiratory complexes (CI and CII) to ROS/NAPQI produced inside the cell following APAP toxicity, thereby causing loss in their activities [67,68,69]. In addition, upon APAP intoxication, oxidation of the mitochondrial GSH pool and MnSOD inactivation by nitration may contribute to the mitochondrial bioenergetics impairment [8,69]. Moreover, APAP or its metabolite NAPQI could directly interact with the inner mitochondrial membrane, causing changes in its fluidity [8] and consequently alterations in the activity of the respiratory chain. Based on this, modifications of the mitochondrial antioxidant system may be responsible for the impairment of oxygen consumption seen herein.

Interventions that restored mitochondrial ROS and peroxynitrite scavenging capacity or prevented mitochondrial permeability transition pore opening with iron chelators have been shown to protect against APAP liver injury [7,9,69]. Recent works have shown that natural antioxidant products such as resveratrol and quercetin can protect against APAP hepatotoxicity through prevention of mitochondrial dysfunctions [70,71]. In this regard, it was found that *Teucruim* species fully preserves mitochondrial respiration and increases GSH levels in cultured HepG2 cells [57]. Besides, it has been reported that *Genista* and *Teucruim* species possess a good antioxidant activity toward a range of free radicals species, with high reducing activity and iron chelating abilities [23,32,57]. We believe that improvement of mitochondrial functionality with *Gq* or *Tp* extract may be linked to a direct stimulation of ROS/NAPQI scavenging ability and/or iron chelating activity by their polyphenolic compounds, which in turn relieved the electron transport chain from the oxidative insult and stabilized mitochondrial membrane fluidity. In contrast to *Tp* extract, *Gq* extract exhibited only a modest protective effect on APAP-induced mitochondrial respiration defect, as observed for improvement of liver histology. Different efficiencies in order to restore mitochondrial activity of *Gq* and *Tp* extracts are probably linked to structure-activity differences of polyphenols contained in each plant to prevent mitochondrial dysfunctions induced by oxidative stress [72]. The contribution of individual phenolics to total antioxidant capacity was generally dependent on their structure and content levels in plant extracts [73]. Therefore, we can presume that different components of *Tp* extract are most critical for the pharmacological hepatoprotective effects than that contained in *Gq* extract to prevent liver mitochondrial damages induced by APAP. Further studies are needed to identify differences in the composition of phenolic compounds in *Gq* and *Tp* extracts, as well as the compared efficiency of each of them to counteract oxidative stress.

The important role of NAC in fully preventing APAP toxicity, oxidative stress, and loss of mitochondrial potential membrane has been previously reported [6]. Except its improvement of state 4 and 3 respiration, NAC failed to protect mitochondrial respiratory complexes activities in the present study. In agreement with our data, another study has shown that NAC does not bring a complete protection of mitochondrial activity in HepG2 cells during Aspirin-induced toxicity [74].

CYP2E1 has been reported to play a dominant role in bioactivation of APAP by conversion to its active hepatotoxic metabolite, NAPQI [75]. In this work, we observed that APAP treatment increased CYP2E1 isoform expression. As observed by Ghosh *et al.* [75], the alteration of the oxidative status previously shown could be aggravated by enhanced formation of reactive oxygen species through cytochrome P450-mediated APAP bioactivation. Interestingly, this increase in CYP2E1 levels was markedly suppressed by *Tp* extract, *Gq* extract or NAC pretreatment. It has been found that drugs with CYP2E1 inhibitory characteristics might possess ability to suppress APAP-induced hepatotoxicity by reducing NAPQI formation [76]. Polyphenols could modulate the cytochrome CYP450 system through the decrease in their hepatic content, the inhibition of their activity and the expression of these enzymes [77]. Consequently, a direct modulation of CYP2E1 activity or levels by polyphenols may favor hepatoprotection against APAP toxicity [78], thus partly explaining the improvement of APAP toxicity induced by *Tp* or *Gq* extract. Finally, inhibition of the CYP2E1 pathway leading to a decrease in NAPQI formation could also explain the restoration of GSH levels and improvement of the oxidative status reflected by improvement of TBARs levels.

The phase II enzyme of drug metabolism GST catalyzes conjugation of toxic electrophiles with glutathione and thereby plays an important role for the detoxication of such metabolites. GSTpi is one of GSTs forms considered as the more effective catalyst of the conjugation of NAPQI with GSH [79]. In the present study, we found that GST activity was reduced whereas GSTpi mRNA levels were found dramatically increased in APAP treated rats. Pretreatment with NAC, polyphenolic extracts from *Gq* or *Tp* significantly enhanced GST when compared to the control APAP treated group. These results are in agreement with a previous study concerning quercetin, curcumin and NAC following APAP intoxication in rats [14]. Interestingly, we observed that the three pretreatments also markedly blunted the increase of GSTpi mRNA induced by APAP. The importance of GSTpi in APAP induced alterations is suggested by several studies. According to Henderson *et al.* humans with higher GSTpi expression are more sensitive to APAP toxicity [80]. In the same line, GSTpi null mice are less sensitive to APAP induced hepatotoxicity, in association with a higher expression of antioxidant proteins [80,81]. Consequently, the decrease in GSTpi expression by *Gq* extract, *Tp* extract or NAC observed in this study is probably involved in the hepatoprotective influence of these extracts against APAP toxicity.

The increase susceptibility to APAP-induced liver injury appeared to correlate with an elevated expression of liver pro-inflammatory cytokines, TNF-α, and IL-1, as well as inducible nitric oxide synthase [82,83]. It was found that gene expression of TNF-α in treated liver was enhanced in a similar pattern as the level of the corresponding protein [84]. In addition, liver TNF-α mRNA expression level has been shown to serve as a valuable indicator for inflammatory response occurrence following APAP toxicity [59]. In agreement with these data, APAP administration significantly up-regulated TNF-α mRNA expression, suggesting the occurrence of a severe inflammatory response, which may be influenced by the concomitant oxidative stress situation. Moreover, pretreatment with *Gq* extract, *Tp* extract or NAC markedly blunted the increase of TNF-α mRNA expression following APAP treatment. These findings are consistent with previous studies demonstrating that NAC decreased TNF-α production and oxidative stress induced by APAP toxicity [83,85]. In the same line, genistein, the most abundant isoflavone compounds in *Genista* species with antioxidant potential [23], was found to block NASH progression through suppression of TNF-α [86]. Furthermore, anti-inflammatory effect of *Teucruim* species has also been reported [66,87], likely linked to the attenuation of JNK activation [66]. In addition, apigenin and luteolin are main antioxidant flavonoids detected in *Teucrium* species [36], with the ability to inhibit TNF-α induced JNK activation during inflammation processes [73]. JNK is thought to play a role in regulating the TNF-α mediated increase of APAP toxicity and its inhibition provide healing liver protection [88,89]. Our data suggest that polyphenolic extracts from *Gq* and *Tp* may exert a part of their anti-inflammatory effects and hepatoprotective influence by decreasing TNF-α expression and consequently, as seen for other antioxidants, by inhibition of the JNK pathway.

However, an interesting question remains to be addressed. It has been shown that NAC administration, the presently more efficient treatment of APAP overdose, needs to be given before or very fast after APAP overdose to overcome hepatotoxicity. This observation suggests that the processes induced by APAP and protected by NAC could be irreversibly altered when fully induced [90,91]. Consequently, it could be interesting to determine the respective ability of Gq and Tp extracts to prevent or restore the consequences of APAP toxicity when given simultaneously or several times after its administration.

## 5. Conclusions

Our study demonstrates, for the first time, *in vivo* hepatoprotective activity of polyphenolic extracts from *Gq* and *Tp*, which attenuates hepatic oxidative stress and reduces transaminases leakage. The protective effects of *Gq* and *Tp* extracts can be mostly attributed to the modulation of mitochondrial bioenergetics, phase I and II enzymes and inflammatory processes upon APAP toxicity. Additional studies performed with the aim to characterize the active principles of our extracts to be used in pharmaceutical, food and nutraceutical industries are currently in progress.

## Figures and Tables

**Table 1:** TLC-fingerprint analysis of *Gq* and *Tp* extracts.

	Neu-Reagent UV/365 nm			
	Band N°	R*_f_*	Color	Type of Phenol/Possible Flavonoid
*Gq*	1	0.03	Blue-white	Flavonols, flavones, isoflavones, flavonones
	2	0.05	Orange	Flavonols glycosides
	3	0.09	Yellow-green	Favonols
	4	0.14	Yellow-pale	Flavonols, flavones, isoflavones, flavonones
	5	0.18	Blue	Phenolic acid
	6	0.26	Blue	Flavonoïd aglycones
	7	0.32	Orange	Flavonols, flavones, isoflavones, flavonones
	8	0.36	Red	Methylated flavones
	9	0.42	Orange	Not identified
	10	0.50	Yellow	Phenolic acid
	11	0.55	Yellow-pale	Phenolic acid
	12	0.78	Blue fluorescent	Phenolic acid
*Tp*	1′	0.05	Blue-white fluorescent	Phenolic acid
	2′	0.12	Yellow-green	Not identified
	3′	0.19	Blue-white	Phenolic acid, isoflavones, flavonones
	4′	0.26	Yellow-orange	Flavonols
	5′	0.31	Yellow-green	Flavonols, flavones, isoflavones, flavonones
	6′	0.36	Orange	Flavonols-glycosides
	7′	0.44	Red	Not identified
	8′	0.54	Blue-white	Not identified
	9′	0.98	Blue-white fluorescent	Phenolic acid

TLC-fingerprint analysis conditions: Eluent: ethyl acetate/ethanol/formic acid/water (100:11:11:26); Support: Merck TLC silica gel 60 F254 and Detection: under UV light in 365 nm after revelation with Neu-reagent (1%). R*_f_*: Retention factor.

**Table 2:** Effect of *Gq* and *Tp* extracts on liver oxidative stress markers following APAP toxicity in rats.

Groups	Control	APAP	NAC+APAP	*Gq*+APAP	*Tp*+APAP
TBARs (nmol/g liver)	53 ± 8	87 ± 13 ***	48 ± 11 ^##^	56 ± 9 ^##^	58 ± 19 ^#^
SOD (U/mg prot)	14 ± 2	11 ± 1 ***	15 ± 1 ^##^	16 ± 2 ^##^	15 ± 1 ^##^
GPx (mU/mg prot)	4192 ± 865	2453 ± 487 ***	4289 ± 398 ^##^	5021 ± 69 ^##^	4594 ± 448 ^##^
GR (mU/mg prot)	102 ± 9	75 ± 12 ***	114 ± 10 ^##^	123 ± 4 ***^,##^	116 ± 8 *^,##^
GSH (nmol/g liver)	1010 ± 235	445 ± 79 ***	930 ± 213 ^##^	807 ± 182 ^##^	923 ± 201 ^##^

Results are expressed as means ± SD (*n* = 6). One-way ANOVA followed by Fisher’s test: * *p* < 0.05, ** *p* < 0.01, *** *p* < 0.001 *vs.* Control group, ^#^ *p* < 0.01, ^##^ *p* < 0.001 *vs.* APAP group. Thiobarbituric acid-reactive substances (TBARS), superoxide dismutase (SOD), glutathione peroxidase (GPx), glutathione reductase (GR), reduced glutathione (GSH).

**Table 3:** Effect of *Gq* or *Tp* extracts on oxygen uptake in state 3 and 4, RCR, respiratory complexes (CI-CV) and citrate synthase (CS) activities in isolated liver mitochondria following APAP toxicity in rats.

Groups	Control	APAP	NAC + APAP	*Gq* + APAP	*Tp* + APAP
State 4 ^§^	110.0 ± 0.8	44.6 ± 6.2 ***	81.9 ± 3.2 ^##^	58.7 ± 7.1	87.5 ± 2.8 ^##^
State 3 ^§^	687 ± 122	136 ± 14.9 **	387 ± 67.1^##^	255 ± 63.7	577 ± 47.8 ^###^
RCR ^@^	6.2 ± 1.1	3.0 ± 0.6 *	4.7 ± 0.7	4.3 ± 0.7	6.6 ± 0.8 ^#^
CI ^$^	112 ± 25	82 ± 10 **	96 ± 15	134 ± 28 ^###^	147 ± 17 **^,###^
CII ^$^	178 ± 43	132 ± 30 *	164 ± 40	271 ± 64 **^,###^	248 ± 23 **^,###^
CII+III ^$^	160 ± 37	145 ± 60	183 ± 31	173 ± 44	200 ± 37
CIV ^$^	749 ± 155	729 ± 155	699 ± 113	1063 ± 184 **^,##^	994 ± 115 **^,##^
CS ^$^	324 ± 39	262 ± 32 **	304 ± 30 ^#^	372 ± 65 ^##^	387 ± 91 ^##^

Results are means ± SD (*n* = 6). One-way ANOVA followed by Fisher’s test: * *p* < 0.05, ** *p* <0.01 and *** *p* < 0.001 *vs* Control group, ^#^ *p* < 0.05, ^##^ *p* < 0.01, ^###^ *p* < 0.001 *vs.* APAP group. ^§^: pmol O_2_/min/mg protein, ^@^: State4/State3, ^$^: mIU/mg protein.
